# Baseline Mapping of Schistosomiasis and Soil Transmitted Helminthiasis in the Northern and Eastern Health Regions of Gabon, Central Africa: Recommendations for Preventive Chemotherapy

**DOI:** 10.3390/tropicalmed3040119

**Published:** 2018-11-11

**Authors:** Rodrigue Mintsa Nguema, Jacques F. Mavoungou, Krystina Mengue Me Ngou-Milama, Modeste Mabicka Mamfoumbi, Aubin A. Koumba, Mariama Sani Lamine, Abdoulaye Diarra, Ghislaine Nkone Asseko, Jean R. Mourou, Marielle K. Bouyou Akotet, Hélène Moné, Gabriel Mouahid, Julienne Atsame

**Affiliations:** 1 Research Institute in Tropical Ecology, National Center for Scientific and Technological Research, Libreville BP 13354, Gabon; mavoungoujacques@yahoo.fr (J.F.M.); aubinho25@yahoo.fr (A.A.K.); 2 Department of Parasitology-Mycology, Faculty of Medicine, University of Health Sciences of Libreville, Libreville BP 4009, Gabon; mabikmamfoumbi@yahoo.fr (M.M.M.); mangondu20@yahoo.fr (J.R.M.); mariellebouyou@gmail.com (M.K.B.A.); 3 Control Program of Parasitic Diseases, Libreville BP 2434, Gabon; ngoukrystina@yahoo.fr (K.M.M.N.-M.); julienneatsame@yahoo.fr (J.A.); 4 National Laboratory of Public Health, Libreville BP 10736, Gabon; 5 Word Health Organization Country Office Gabon, Libreville BP 820, Gabon; salamarine_06@yahoo.fr (M.S.L.); diarraa@who.int (A.D.); nkoneassokog@who.int (G.N.A.); 6 IHPE, Univ Montpellier, CNRS, UM, IFREMER, Univ Perpignan Via Domitia, Perpignan F-66860, France; mone@univ-perp.fr (H.M.); mouahid@univ-perp.fr (G.M.)

**Keywords:** schistosomiasis, soil-transmitted-helminthiasis, mapping, preventive chemotherapy, transmission control, Gabon, Central Africa

## Abstract

In order to follow the Preventive Chemotherapy (PC) for the transmission control as recommended by WHO, Gabon initiated in 2014 the mapping of Schistosomiasis and Soil Transmitted Helminthiasis (STH). Here, we report the results of the Northern and Eastern health regions, representing a third of the land area and 12% of its total population. All nine departments of the two regions were surveyed and from each, five schools were examined with 50 schoolchildren per school. The parasitological examinations were realized using the filtration method for urine and the Kato-Katz technique for stool samples. Overall 2245 schoolchildren (1116 girls and 1129 boys), mean aged 11.28 ± 0.04 years, were examined. Combined schistosomiasis and STH affected 1270 (56.6%) with variation between regions, departments, and schools. For schistosomiasis, prevalence were 1.7% across the two regions, with no significant difference (*p* > 0.05) between the Northern (1.5%) and the Eastern (1.9%). Schistosomiasis is mainly caused by *Schistosoma haematobium* with the exception of one respective case of *S. mansoni* and *S. guineensis*. STH are more common than schistosomiasis, with an overall prevalence of 56.1% significantly different between the Northern (58.1%) and Eastern (53.6%) regions (*p* = 0.034). *Trichuris trichiura* is the most abundant infection with a prevalence of 43.7% followed by *Ascaris lumbricoides* 35.6% and hookworms 1.4%. According to these results, an appropriate PC strategy is given. In particular, because of the low efficacy of a single recommended drug on *T. trichiura* and hookworms, it is important to include two drugs for the treatment of STH in Gabon, due to the high prevalence and intensities of *Trichuris* infections.

## 1. Introduction

Combined schistosomiasis and Soil Transmitted Helminthiasis (STH) are the most prevalent infectious diseases in the world. They are the cause of serious global public health problems and impose a great burden on poor populations in the developing world [1]. Indeed, with the exclusion of malaria, the World Health Organization (WHO) further estimated that schistosomiasis and STH were responsible for more than 40% of the disease burden due to tropical diseases [2].

Schistosomiasis is acknowledged to be distributed in Africa, Asia, and South America with about 200 million infected people [3]. The WHO regards the disease as a Neglected Tropical Disease (NTD), with an estimated 732 million persons being vulnerable to infection worldwide in renowned transmission areas [4]. The Sub-Sahara African region remains the most affected with still high prevalence with about 192 million people infected [5]. In Gabon, with confirmed occurrence of *Schistosoma guineensis*, formely *S. intercalatum* lower Guinea strain, [6,7] and *S. haematobium*, with some cases of *S. mansoni*, it is estimated that a total of 310,391 people require preventive chemotherapy (PC) [8]. However, these 2010 estimates likely need revising.

Soil Transmitted Helminthiasis corresponds to a group of parasitic diseases that are caused by nematode worms that are transmitted to humans by faecally contaminated soil. The three major human diseases are caused by *Ascaris lumbricoides*, *Trichuris trichiura*, and hookworms (*Necator americanus* and *Ancylostoma duodenale*). The latest estimates indicate that more than two billion people are infected by at least one species worldwide and more frequently in areas where sanitation and water supply are insufficient [9]. STH infections are most common in children and such children have malnutrition, growth stunting, intellectual retardation, and cognitive and educational deficits [10]. It is estimated that over 35.4 million African school-aged children (SAC) are infected by *A. lumbricoides*, 40.1 million with *T. trichiura*, and 41.1 million with hookworms [11]. Since many children have multiple infections, it is estimated that 89.9 million are infected by at least one STH species [11]. In 2009, according to the burden of STH per country in the WHO African Region, 145,518 preschool (1−5 years) and 349,386 SAC (6−14 years), with a need of revising, were requiring PC in Gabon [9]. Indeed, the situation of STH in Gabon remains of concern and the latest local studies that were carried out showed that the prevalence is low in the sub-urban area (Melen, Libreville) and moderate in the rural area (Ekouk, 80 km of Libreville) [12].

Despite the burden that they cause in world public health, schistosomiasis and STH are considered as NTD’s. Since the World Health Assembly in 2001, access to essential medicines for schistosomiasis and STH in endemic areas for the treatment of both clinical cases and groups at high risk for morbidity was recommended and endorsed by World Health Assembly resolution WHA54.19. The resolution urges member states to ensure access to essential drugs against schistosomiasis and STH in all health services in endemic areas for the treatment of clinical cases and groups at high risk of morbidity such as women and children. The declared that the aim of this resolution was to achieve at least the 75% coverage target of regular administration of anthelminthic drugs and up to 100% of all SAC at risk of morbidity by 2010 [13]. The strategy that was adopted by WHO since 2006 advocates integrated PC using a school-based approach with the concept of coordinated use of anthelminthic medicines against schistosomiasis and STH given the consideration that the diseases are largely co-endemic and that these medicines can safely be co-administered [14]. The 2010 target was not achieved, only 200 million SAC of the 600 million in need received treatment in 2010 [9]. The current goal is to revitalize the control strategy for achieving the 75% coverage target by 2020. PC for the populations at-risk in endemic areas was adopted once or twice a year, depending on risk levels, over a five-year period. Preschool and SAC in endemic areas were the primary target of PC interventions. Therefore, the target population was expanded to include all adults in high-risk areas. Communities can be classified into low-risk (<10% for schistosomiasis and <20% for STH), moderate-risk (≥10% for schistosomiasis and ≥20% for STH but <50% for both), and high-risk (≥50% for both) areas. These prevalence based on the SAC sampling are essential to adapt the frequency of PC (including SAC and at risk adults in the whole communities) according to the WHO disease specific thresholds [15].

Thus, the first step for establishing the PC strategy for schistosomiasis and STH is the knowledge of the geographic distribution of prevalence and the degree of overlap of the diseases in endemic areas [16]. The distribution of schistosomiasis and STH is particularly sensitive to environmental changes whose heterogeneity reflects numerous human and ecological factors, including changes of human origin and focal transmission [17]. For these purposes, there is a need to identify restricted areas where infection remains a public health problem for an integrated control identifying the broad scale patterns. A successful role for GIS applications in investigating the spatial epidemiology of the major human helminths was well recognized and helping to this purpose [18]. In 2009, 20 of the 32 African endemic countries had initiated the mapping of schistosomiasis and STH in order to implement the PC interventions [9]. In Gabon, schistosomiasis and STH are known to occur in many areas [8,9]; however, there has not been any sustained effort to control the diseases, apart from the establishment of the National Program for Control of Parasitic Disease in 1999, and to date, no major action had been taken at the national level by this program.

Until early 2014, PC interventions for schistosomiasis and STH had not been started in Gabon. To be in line of the WHO’s target for the control of schistosomiasis and STH, Gabon initiated, in April 2014, the evaluation of the prevalence levels of schistosomiasis and STH throughout its territory. The aim was to report the outcome of schistosomiasis and STH at several levels in order to provide recommendations that are related to the implementation of PC interventions according to the WHO requirement. The present paper publishes the results regarding the Northern and Eastern health regions of Gabon.

## 2. Materials and Methods 

### 2.1. Authorization and Ethical Assessment

Gabon aligns with the NTD coordinated mapping guidelines of WHO [15]. The implementation of the present study will enable the rapid scaling up of national mass treatment interventions and achieve the WHO targets that are set for 2020. An agreement was obtained for the implementation of this study as a public health exercise assumed for the Ministry of Health. Surveys were conducted in schools with the approval of the Ministry of National Education, school inspectors, directors, and teachers. Informed written consent was sought from the directors of selected schools as the legal guardian of all study schoolchildren. The school director receives prior verbal consent of the parents or guardians of schoolchildren after having explained to them the study and its objectives, with a translation in local language when necessary. Each individual that was involved in the study was registered in a data file and assigned to an anonymous identification number. At the end of the trial, infected persons were provided with appropriate chemotherapy, before the beginning of PC interventions, according to the WHO recommendations.

### 2.2. Study Area

**Figure 1:**
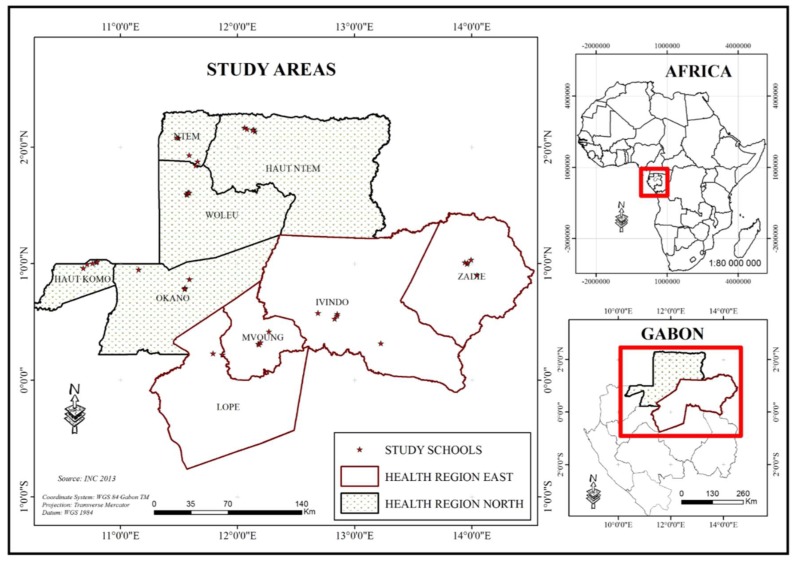
Study area showing the nine health departments surveyed: in the Northen region (gray): Woleu, Ntem, Haut-Ntem, Haut-Okano, Okano and in the Eastern region (white): Ivindo, Lopé, Mvoung, and Zadié.

Gabon is part of Central Africa. The Ministry of Public Health divides the country into 10 health regions and 52 departments. The health regions analyzed in this paper represent a third of the land of Gabon and include nine health departments, with five (Woleu, Ntem, Haut-Ntem, Haut-Okano and Okano) in the North region and four (Ivindo, Lopé and Mvoung and Zadié) in the East region (Figure 1). The General Direction for Statistic (GDS) estimated the total Gabonese population at 1,811,079 inhabitants, of which 34.7% were pre and SAC, and an urbanization rate of 87% [19]. However, predominantly urban population live in only 1% of the total space of the country, whereas the majority of territory (99% of superficies) is rural and hosts only 13% of population of the county. The study area comprises a total population of 218,279 inhabitants (154,986 and 63,293 in the North and East health regions, respectively).

### 2.3. Study Type, Period and Population

A cross-sectional prospective study was carried out from January to February 2015. It included both male and female schoolchildren aged 10 to 14 years in priority (where the infection rates will be the highest and where WHO base their guidelines from) in the selected schools. Each included schoolchild must have provided both stool and urine, otherwise they were excluded from the survey.

### 2.4. Selection and Location of Schools

Each health department was considered as an ecologically homogeneous area. Five schools were selected randomly among those available for each department. Schools were either urban or rural and either public or private. The geographical coordinates of each school were recorded while using a Garmin GPS (Global Positioning System) (Table S1).

### 2.5. Schoolchildren Sampling

In each school, select 50 schoolchildren (25 girls and 25 boys) aged 10 to 14 years from the upper class, for a total of 250 schoolchildren in each health department. For the sampling, align all schoolchildren in the age group of interest from the upper class in two rows according to their gender (girl and boy). In each row (gender), select 25 schoolchildren.

If the row contains more than 25 schoolchildren, then a systematic random sampling method is used. For example, say there are 100 schoolchildren in the row, divide the total number of schoolchildren (100) by the number of schoolchildren you want in the sample (25), the answer is 4. This means that you are going to select every fourth schoolchildren from the row. Choose randomly a number between 1 and 4. This is your random starting point. Say your random starting point is “3”, this means you select schoolchild 3 in the row as your first schoolchild, and then every fourth schoolchild down the row (3, 7, 11, 15, 19, etc.) until you have 25 schoolchildren.

If the row contains exactly 25 schoolchildren, no random sampling is necessary; all 25 schoolchildren are directly selected.

If the row contain less than 25 schoolchildren, select them and complete the sample with schoolchildren in the age group of interest from the next upper class using a random sampling method, as described above. 

### 2.6. Sample Collection and Parasitological Examination

Stool and urine samples of each individual were collected from 9.00 to 11.00 h am in a 100 and 50 mL of plastic screw-cap vial respectively and forwarded for examination in the laboratories equipped for the circumstance at the department level. Small cakes have been distributed to encourage them. Those who did not provide both samples despite any efforts were replaced according to the schoolchildren sampling protocol. For schools with fewer than 50 schoolchildren, enrollment is completed among the other schools that were selected in the same district. For each selected schoolchild, the urine and stool samples were collected, along with information on gender and age.

Urine was analyzed for the presence and the number of *S. haematobium* eggs, using a slightly modified Nucleopore syringe urine filtration method [20], filtering a 10 mL unique aliquot from each urine sample [21]. When the volume of the sample was less than 10 mL, it was measured before filtration and the number of eggs per 10 mL was estimated. Intensity of *S. haematobium* infection was expressed as the number of eggs per 10 mL of urine (eggs/10 mL). Stool samples were examined for the presence and the number of both STH and intestinal *Schistosoma* (*S. mansoni* and *S. guineensis*) eggs while using the Kato-Katz technique [22]. A single thick smear equivalent to 41.7 mg of stool was analyzed for each stool sample. The method used is that described in the Kato-Katz kit (VESTERGAARD FRANDSEN). Eggs were immediately examined and counted by microscopy to avoid egg lysis of hookworm eggs. Individual intensity of infection was expressed as eggs per gram of feces (epg).

### 2.7. Data Analysis

All the collected data: age, gender, and parasitological results, were reported on an Excel sheet. Prevalence of infection (percentage of infected schoolchildren among those examined) was estimated for each parasite, for each parasite group: schistosomiasis and STH and for the combined schistosomiasis and STH, at the overall, regional, departmental, school, school category (public/private), school location (urban/rural), and gender levels. The 95% confidence intervals (CI) for prevalence were calculated using the exact method in the software R version 3.2.2. Arithmetic mean intensities of infection (number of egg per infested schoolchild) with standard deviations (SD) for each parasite species were estimated, including only the positives schoolchildren [23]. The Chi squared or Fisher exact tests were used to compare the prevalence differences in relation to the region, gender, school category (private/public), and school area (urban/rural), while the non-parametric Wilcoxon or Mann-Whitney rank sum test were used to compare differences in mean intensities of infection using R version 3.2.2 or SPSS 10.0 for Windows software. The significance of tests was defined at *p* < 0.05. Prevalence generated in each department were used to produce the prevalence maps of distribution for each species using software ArcGis version 10.1.

## 3. Results

### 3.1. Characteristics of Sampling 

A total of 45 schools were examined, 25 for North and 20 for East region, 27 for urban versus 18 for rural area and 27 for public versus 18 for private category. A total of 2245 schoolchildren (1116 girls and 1129 boys) and the mean number of schoolchildren per school was 49.9 ± 3.9. The number of examined schoolchildren is 1236 (632 girls and 604 boys) in the North region and 1009 (484 girls and 525 boys) in the East region. 1754 schoolchildren were from urban versus 491 from rural area and 1420 public versus 825 private. Age of schoolchildren ranged from 4 to 17 years with median age of 11 years. The total average ages of examined schoolchildren were 11.28 ± 0.04; 11.39 ± 0.05 in the Northern and 11.26 ± 0.09 in the Eastern region.

### 3.2. Prevalence

#### 3.2.1. Combined Schistosomiasis and Soil Transmitted Helminthiasis

Of the 2245 schoolchildren surveyed, 1270 (56.57%; 95% CI 54.49–58.63%) were affected by schistosomiasis (at least one species) and/or STH (at least one species) (Table 1). Schoolchildren from the North region: 723 (58.50%; 95% CI 55.3–60.9%) were significantly more infected than those from the East region: 548 (54.31%; 95% CI 50.5–56.7%), (X-squared=4.5129, df = 1; *p* = 0.04169). At the department level, prevalence was from 44.4% (WLE department) to 73.6% (HKO department) in the Northern region and from 46.56% (LPE department) to 67.45% (ZAD department) in the Eastern. Prevalence was significantly different between departments (X-squared = 84.672, df = 8, *p* < 0.0001). All of the schools were infected with prevalence ranging from 28.6% at school 3 of WLE to 92.9% at school 3 of NTM in the North region and from 8.3% at school 4 of MVG department to 88.2% at school 4 of IVD in the East region (Table S2). There were significant differences between schools (X-squared = 325.31, df = 44, *p* < 0.0001). Gender and school category had no influence on prevalence of the combined schistosomiasis and/or STH (*p* > 0.05), while STH infections in rural schoolchildren (72.10%; 95% CI 67.90–76.02%) were significantly more prevalent than the urbans (51.60; 95% CI 49.23–53.96%), (X-squared = 64.633, df = 1, *p* < 0.00001). 

Of the total 1270 affected schoolchildren, 718 had one species, 537 two species, 15 three species, and no schoolchild had four, five, six or more parasites concomitantly. For those affected with schistosomiasis only one had two species, and for those with STH, 528 had two species, eight had three species, and the rest had one species (Table 2).

#### 3.2.2. Combined Schistosomiasis

Including all three species, overall 38 cases (1.7%) were positive for schistosomiasis in 21 schools of the 45 surveyed, with no significant difference (*p* > 0.05) between the North, 19 cases (1.5%; 95% CI 0.9–2.4%), and the East region, 19 cases (1.88%; 95% CI 1.1–2.9%), (Table 1). Gender, school area and school category had no influence on prevalence of the combined schistosomiasis (*p* > 0.05). At the department level, prevalence was all <10%. It varied from 0.8% at WLE to 2.6% at HKO department in the North region (*p* > 0.05) and from 0.0% at ZAD to 4.4% at MVG department in the East region (X-squared = 22.032, df = 8, *p* = 0.004857). At the school level, prevalence was all < 10% (0% in 25 schools), with the exception of school 5 in MVG department, it was 15.8% (X-squared = 84.762, df = 44, *p* = 0.0002171).

#### 3.2.3. Schistosomiasis Haematobium

**Figure 2:**
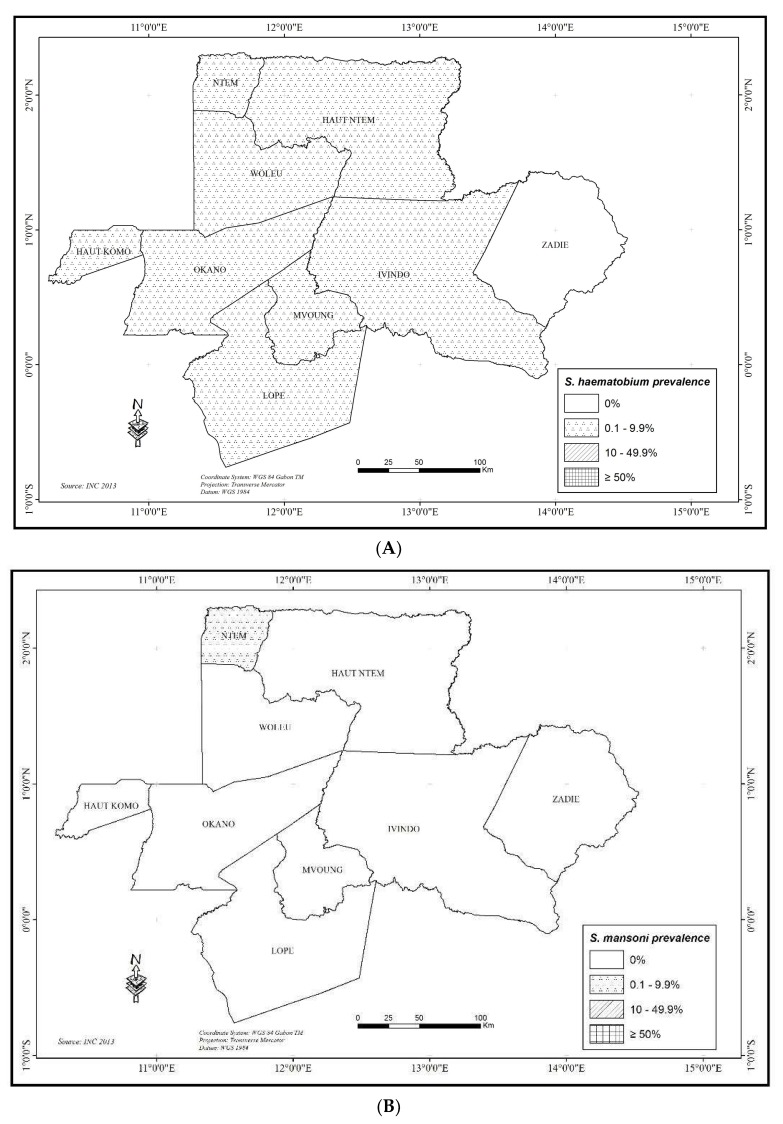

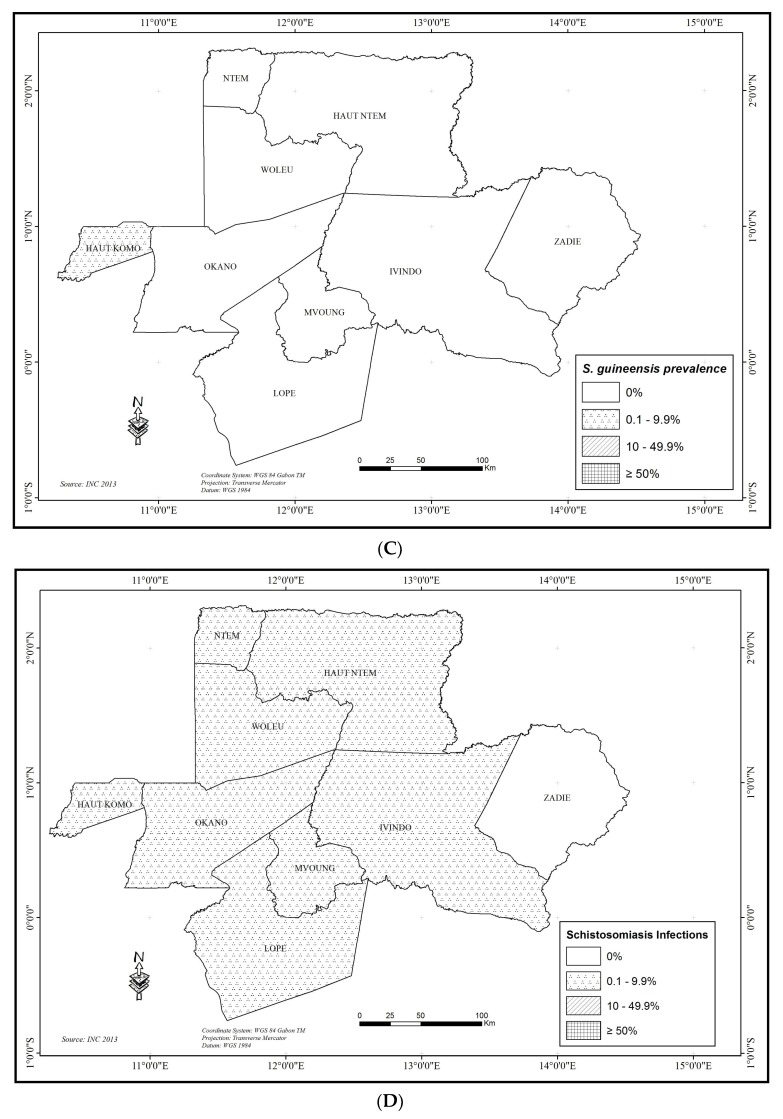
Map of prevalence levels for Schistosomiasis at department scale: (**A**) *Schistosoma haematobium*; (**B**) *S. mansoni*; (**C**) *S. guineensis;* and, (**D**) Combined schistosomiasis.

It is the most frequent schistosomiasis that was found to be prevalent in 20 schools from the 45 studied (Table S2). Overall, *S. haematobium* affected 37 (1.45%; 95% CI 1.16–2.27%) schoolchildren with 18 (1.46%. 95% CI 0.87–2.29%) in the North and 19 (1.88%; 95% CI 1.14–2.93%) in the East region (*p* > 0.05). Gender, school area, and school category had no influence on prevalence of *S. haematobium* (*p* > 0.05). At the department level (Figure 2), the prevalence of *S. haematobium* was all <10%, from 0.8% (HNT department) to 2.4% (NTM department) in the North region and from 0% (ZAD department) to 4.36% (MVG department) in the East region, (X-squared = 21.741, df = 8, *p* = 0.00542). At the school level, prevalence varied from 0 (in 25 schools) to15.8% (six cases in school 5 in the MVG department) (X-squared = 85.959, df = 44, *p* = 0.0001583).

#### 3.2.4. Other Schistosomiasis

*S. guineensis* and *S. mansoni* are very unusual, only one case of each respectively was listed in the North region. The *S. mansoni* case was encountered in school 4 of NTM department, while the *S. guineensis* case was in school 1 of HKO department (Table S2). All the distribution maps of schistosomiasis are presented in the Figure 2. 

#### 3.2.5. Combined Soil Transmitted Helminthiasis (STH)

Including all the STH, a total of 1259 (56.08%; 95% CI 54.0–58.15%) schoolchildren were affected by STH (Table 1). At the regional level, the North health region (58.09%; 95% CI 55.29–60.86%) was more affected than Eastern (53.62%; 95% CI 50.48–56.73%), (X-squared = 4.3331, df = 1, *p* = 0.03738). At the department level, significant differences were found (X-squared = 85.435, df = 8, *p* < 0.0001). Prevalence varied from 44.736% (WLE department) to 73.16% (HKO department) in the North and from 46.56% (LPE department) to 67.45% (LPE department) in the East region. At the school level, there were heterogeneity between schools (X-squared = 326.25, df = 44, *p* < 0.00001). One school had a prevalence of STH < 20%, 14 schools had a prevalence ≥20%, but <50% and 29 had a prevalence ≥50% (Table S2). Gender and school category had no influence on overall prevalence of STH (*p* > 0.05) while according to school area: schoolchildren of rural schools (72.1% [67.90–76.02]) had significantly higher prevalence than those of urban schools (51.6%; 95% CI 49.23–53.96%), X-squared = 64.633, df = 1, *p* < 0.0001.

#### 3.2.6. *Ascaris lumbricoides*

**Figure 3:**
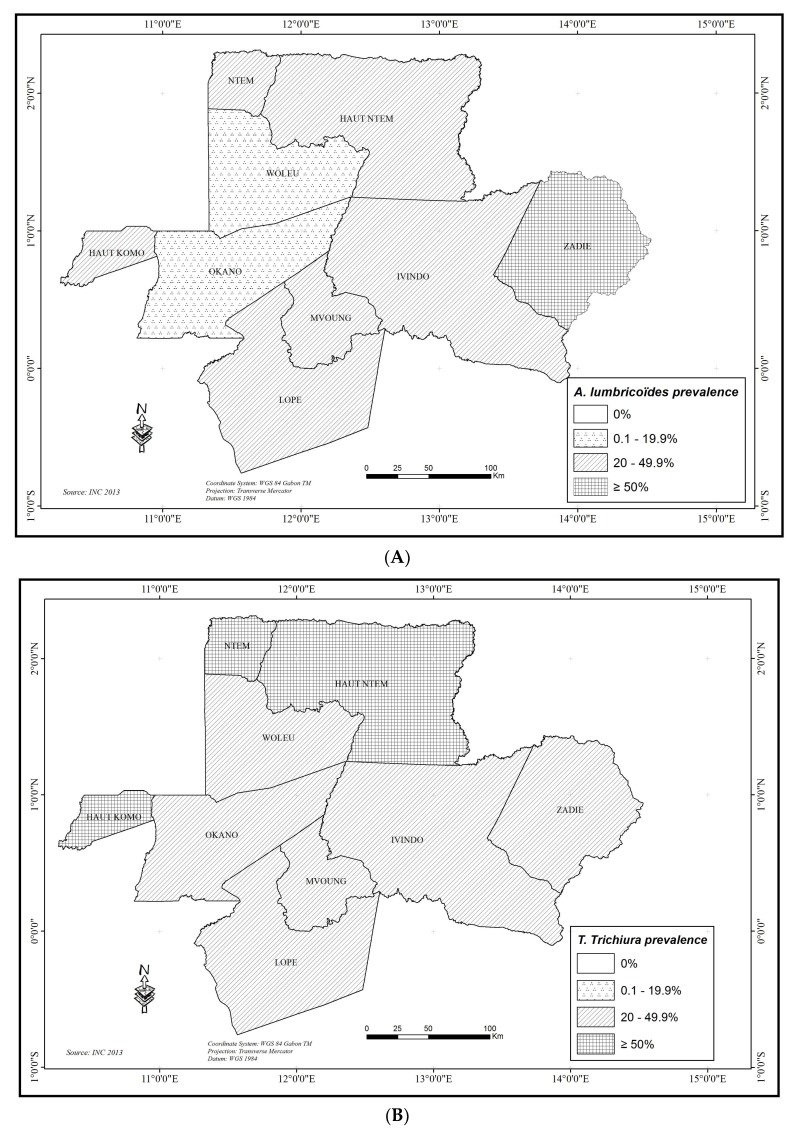

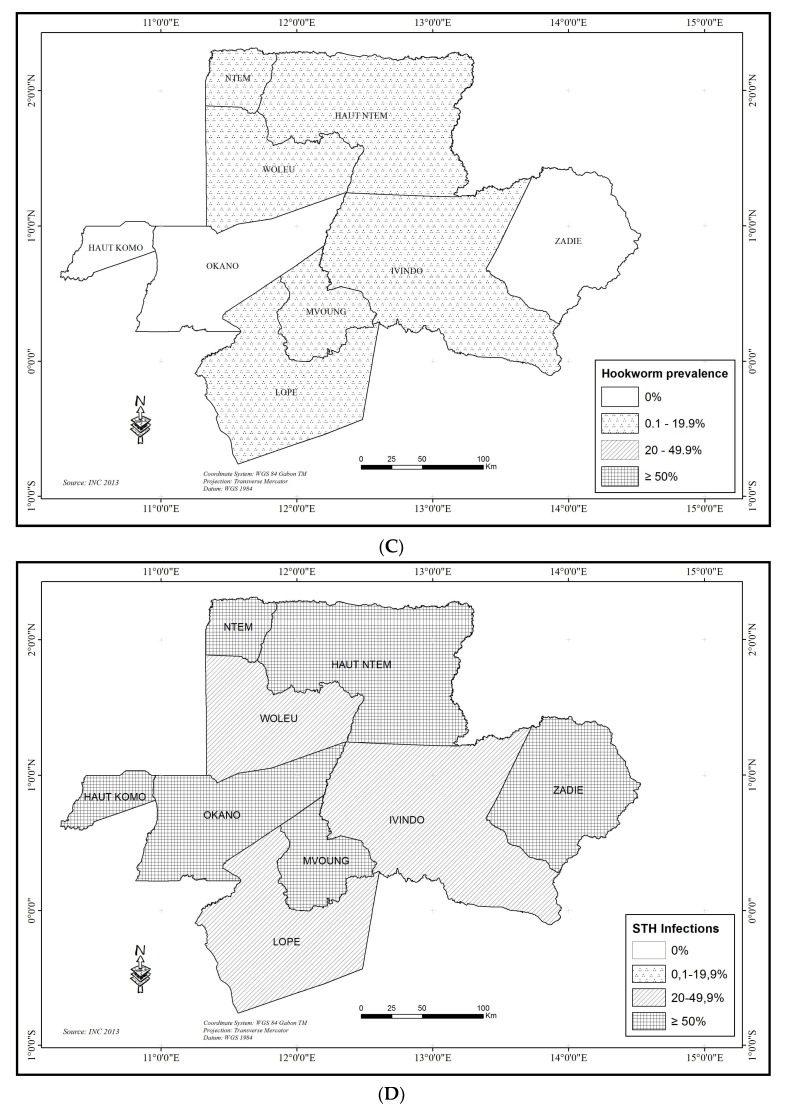
Map of prevalence levels for Soil Transmitted Helminthiasis at the department scale: (**A**) *Ascaris lumbricoides*; (**B**) *Trichurus trichuira*; (**C**) Hookworm; and, (**D**) Combined STH.

*A. lumbricoides* was identified in 44 of the 45 schools studied for a total of 799 (35.59% (95% CI 33.61–37.59%)) schoolchildren affected (Table 1). According to the health region, East (43.51% (95% CI 40.42–46.63%)) was more affected than North (29.13% (95% CI 26.60–31.75%)), (X-squared = 50.126, df = 1, *p* < 0.0001). Gender and school category had no influence on overall prevalence of *A. lumbricoides* (*p* > 0.05) while rural schools (52.55% (95% CI 48.02–57.04%)) were more affected by *A. lumbricoides* than urban schools (30.84% (95% CI 28.69–33.06%)), (X-squared = 77.872, df = 1, *p* < 0.0001). At the department level (Figure 3), prevalence ranged from 12.9% in the WLE department to 58.04% in ZAD, (X-squared = 160.82, df = 8, *p* < 0.0001). Significant heterogeneity (X-squared = 382.81, df = 44, *p* < 0.0001) existed between schools: one is non-infected (0%), nine schools had a low prevalence (<20%), 19 had a moderate prevalence (≥20% but <50%), and 16 had a high prevalence (≥50%) (Table S2).

#### 3.2.7. *Trichuris trichiura*

*T. trichiura* was prevalent in all schools surveyed and it was the more frequently found species with a total of 982 (43.7% (95% CI 41.68–45.8%)) infected schoolchildren. According to health region, a higher prevalence was found in the North (52.8% (95% CI 50–55.7%)) as compared to the East (32.6% (95% CI 29.7–35.6%)), (X-squared = 91.521, df = 1, *p* < 0.0001). There was a significant difference between rural (55%; 95% CI 50.5–59.5%) and urban schools (40.6%; 95% CI 38.3–42.9%), (X-squared = 31.729, df = 1, *p* < 0.0001)); and, between public (45.4%; 95% CI 43.7–48%) and private schools (41%; 95% CI 37.6–44.4%), (X-squared = 3.8965, df = 1, *p* < 0.0001) for the overall prevalence of *T. trichiura*. There was no significant difference of overall prevalence of *T. trichiura* according to gender (*p* > 0.05). At the department level, (Figure 3) the prevalence of *T. trichiura* varied from 27.13% in LPE to 67.53% in HKO department with significant heterogeneity (X-squared = 142.85, df = 8, *p* < 0.0001). At the school level, six schools had a prevalence of *T. trichiura* <20%, 21 schools had a prevalence ≤20% but <50%, and 18 had a prevalence ≥50% (Table S2). There was a significant difference in the distribution of *T. trichiura* among schools (X-squared = 340.14, df = 44, *p* < 0.0001).

#### 3.2.8. Hookworms

Hookworms were present in 12 of the 45 studied schools with an overall prevalence of 1.43% (95% CI 1–2%): 1.6% (95% CI 0.99–2.49%) in the North region, 1.2% (95% CI 0.6–2.1%) in the East region. There was no significance difference between regions, school areas, and school categories (*p*>0.05), while there was a significant difference in overall prevalence of hookworm between girls (1.3%; 95% CI: 0.8–2.2%) and boys (2%; 95% CI: 1.3–3%) (X-squared = 5.2061, df = 1, *p* = 0.02251). At the department level (Figure 3), the prevalence was from 0 (for three departments) to a maximum of 6% in the WLE department with significant difference between departments (X-squared = 53.552, df = 8, *p* < 0.0001). Prevalence of hookworm in schools ranged from 0% (33 of the 45 schools surveyed) to 14.3% (Table S2). There were significant differences between schools (X-squared = 159.25, df = 44, *p* < 0.0001). Geographical distribution of the STH is presented in Figure 3.

### 3.3. Intensity of Infection 

#### 3.3.1. Schistosomiasis

For the 37 schoolchildren that were infected with *S. haematobium*, the mean intensity of infections was 101.9 ± 41.1 eggs per 10 mL of urine with a significant difference between the North (18.3 ± 8.2 epg) and the East region (181.1 ± 85.6 epg), (W = 246, *p* = 0.02176) (Table 3). On the 20 schools prevalent with *S. haematobium*, light-intensity infections (<50 eggs/10 mL) occurred in 12 schools (11 in the North region and one in the East Region) and heavy-intensity infections (≥50 eggs/10 mL) occurred in eight schools (one in the North region and seven in the East Region) (Table S3). The maximum individual egg counts was 1534 eggs/10 mL of urine; 73% of infected schoolchildren had low-intensity infections and 27% had heavy-intensity infections. Overall, there is no significant difference between the genders, the school areas, and the school categories (*p* > 0.05).

Intensity of infections was 72 epg for the only case of *S. mansoni* and 240 epg for the only case of *S. guineensis* (Table 3).

#### 3.3.2. *Ascaris lumbricoides*

Overall mean intensity of infection was moderate: 9586.6 ± 618.3 epg and significantly different between the two regions: 11,433.6 ± 1061.7 epg for the North and 8071.9 ± 707.3 epg for the East region (W = 69,804, *p* = 0.004523) (Table 3). Of the 44 schools prevalent with *A. lumbricoides*, light-intensity infections (1–4999 epg) occurred in 10 schools (four in the North region and six in the East region), moderate-intensity infections (5000–49,999 epg) occurred in 33 schools (20 in the North region and 13 in the East region), and heavy-intensity infections (≥50,000 epg) occurred in one school (in the North region) (Table S3). The maximum individual egg counts was 176,640, whereas 59.1% of infected schoolchildren had low-intensity infections, 37.5% with moderate-intensity infection, and 3.4% with heavy-intensity infections. Overall, gender, school area, and school category had no influence on the *A. lumbricoides* intensity of infection (*p* > 0.05).

#### 3.3.3. *Trichuris trichiura*

The overall mean intensity of infection was moderate: 1143.2 ± 97 with a significant difference between the North (1395.2 ± 126.6 epg) and the East region (642.9 ± 140.2 epg), (W = 76,502, *p* = 1.551 × 10^−13^) (Table 3). Intensities of infection were classified in the light-intensity infections class (1–999 epg) for 30 schools (13 in the North region and 17 in the East region), in the moderate-intensity infections class (1000–9999) for 15 schools (12 in the North region and three in the East region). No school had the heavy intensity infections (≥10,000 epg) (Table S3). The maximum individual egg count was 37,440; 77.8% of the infected schoolchildren had low-intensity infections, 20.1% moderate-intensity infections, and 2.1% heavy-intensity infections. Overall, gender and school area had no influence on the *T. trichiura* intensity of infection (*p* > 0.05), while intensities of infection were higher in public schools (1193.5 ± 113.1 epg) than in private schools (1047.3 ± 182.1 epg) (W = 98,024, *p*-value = 0.01032) in the same class of intensity.

#### 3.3.4. Hookworm

The overall mean intensity of infection was light: 618.0 ± 499.6 epg; 130.8 ± 31.1 epg in the North region and 1430.0 ± 1369.1 epg in the East region (Table 3). No significant difference was found between regions, gender, school areas, and school categories. The maximum individual egg count was 15,840 eggs and 96.9% of the schoolchildren had light-intensity infections (Table S3).

### 3.4. Community Diagnosis and Recommended Treatment Strategies

According to our results on the prevalence and the intensity of infection, the recommended treatment strategies by department were summarized in Table 4.

## 4. Discussion

Our study showed that schistosomiasis and STH remain common in schoolchildren of both North and East health regions of Gabon with heterogenic proportions. Of the 2245 examined schoolchildren, 1270 (56.6%) were diagnosed by at least one schistosomiasis and/or STH. Infections were more influenced by both regions and school area. Indeed, schoolchildren in the North region (58.5%) were more affected than those in the East region (54.2%) and rural schoolchildren (72.10%) were more affected than urban schoolchildren (51.6%). Gender (girl/boy) and school category (public/private) had no influence on the burden of combined schistosomiasis or combined STH. However, hookworms affect more the boys than the girls and *T. trichiura* affect more the public than the private schoolchildren burden was most supported by STH than by schistosomiasis that is very low. 

For schistosomiasis, the present study indicates that the infection is low endemic in the surveyed area, with the prevalence being 1.7% (all three species). Exhaustive results indicate that distribution of schistosomiasis is heterogeneous with an overall low endemicity for all the three species in the whole of the study area. Schistosomiasis haematobium was the most frequent and was diagnosed with at least one case in 20 schools from the 45 studied and from these 20 schools only one school was moderately endemic with a prevalence at 15.8%. Overall infection of *S. haematobium* was low (1.7%) in both the North (1.5%) and the East regions (1.9%). At the departmental level, prevalence was from 0.8% to 2.4% in the North region and from 0 to 4.4% in the Eastern region. Schistosomiasis *mansoni* and *guineensis* were rare in the surveyed areas with only one case of *S. mansoni* and *S. guineensis* respectively from the total schoolchildren examined. Data obtained here contrast with the results available for other areas and for the overall estimations in Gabon. Indeed, Mintsa *et al*. reported prevalence for *S. haematobium* at 17% and 26% in Melen, Libreville and Ekouk (80 km to Libreville), respectively [24], Gabon. Even wider, the estimation of prevalence of schistosomiasis in Gabon was about 45% [3]. Outside Gabon, in Central Africa, prevalence of schistosomiasis is generally high. For instance, in Cameroon, some localities in the East, West, and Central regions had prevalence of between 20 and 50%, and for some of them >50% [25]. In the Littoral, North-West and South-West Cameroon regions [26], prevalence were also much higher than those recorded in our study. This contrast confirms the patching distribution of schistosomiasis. Some parameters can explain the patching distribution of schistosomiasis and they include human and ecological factors [27], temperature, and rainfall [28]. The use of GIS for epidemiological survey in Tanzania showed that schistosomiasis was not endemic in areas where the temperature was below 20 °C [18,29]. By contrast, in Cameroon, prevalence is >10% for the areas where temperature is >40 °C and precipitation <1500 mm [30]. These differences can be attributed to both distribution of the intermediate snail host species in Africa [31] and their optimal conditions for development in West Africa [32]. In our study sites, temperature is >30 °C and precipitation >1000 mm, this should be in favor of high prevalence. Besides temperature and rainfall, relief [33], demography and living conditions [34] can also play a role in the distribution of schistosomiasis. Otherwise, the low presence *S. guineensis* can also be attributed to the possibilities of hybrid species which are mentioned in the country and are very indistinguishable using microscopy [24]. The hypothesis of the hybridization zones between *S. guineensis* and *S. haematobium* has been suggested in two provinces of western Gabon, Moyen-Ogooué [35] and Estuaire [36]. Hydridization between *S. guineensis* and *S. haematobium* led to the extinction of *S. guineensis* in favor of *S. haematobium*, as at Loum in Cameroon [37]. The low prevalence of *S. mansoni* in this study is not surprising because its distribution in the country is uncertain [38].

In addition to the low prevalence recorded in this study, schistosomiasis was characterized by low intensity of infections. Indeed, 73% of schoolchildren that were infected by *S. haematobium* had a light (<50 eggs/10 mL urine) intensity of infection, and 27% a heavy one (≥50 eggs/10 mL urine). These results are lower than those that were recorded at baseline results in the Barombi Kotto focus, Cameroon where the total intensity of infection was 212.1 e/10 mL urine in schoolchildren of ages between 3 and 22 years [23] and in the Sahel region, Burkina Faso [39]. The intensity of infection to *Schistosoma* is often correlated to the morbidity in SAC and other susceptible groups [40,41] and it plays an important role for the estimation of prevalence with consequences for the treatment strategy in PCT [38,42]. Although the microscopic techniques that were used in our study (urine filtration for *S. haematobium* diagnosis and Kato-Katz for *S. mansoni* and *S. guineensis* diagnosis) are the most recommended by WHO [43] and the most widely used diagnostic approaches in epidemiological surveys, their sensitivity is very discussed in foci with low intensity of infection because of day to day egg variations [44]. Hence, multiple Kato-Katz thick smears are required to enhance sensitivity [45], but this poses operational challenges and strains financial resources. As an alternative to these conventional diagnostic methods, novel tools showing a very high diagnostic accuracy have recently been developed. They include the detection of monoclonal antibody-based circulating antigens CCA and CAA [46] and the molecular approaches [47]. For example, it has been shown that estimation of prevalence with Kato-Katz technique underestimates the prevalence of active *S. japonicum* infections in China by a factor of 10 compared with the UCP-LF CAA assay [48]. Similarly, estimation of *S. haematobium* prevalence was several-fold higher with UCP-LF CAA assay than the one detected with a single urine filtration [49]. Since 2008, a more sophisticated Point-Of-Care (POC) test detecting *Schistosoma* CCA in urine has been developed and is now commercially available and is recommended by the authors for *S. mansoni* diagnosis [50,51]. A POC-CCA test revealed higher sensitivity than triplicate Kato-Katz, and it produced similar prevalence as nine Kato-Katz in many field survey evaluations [21,52]. The use of CCAs or CAAs might thereby affect the results and the recommendations for treatment strategies

Our results showed that STH were highly endemic. Overall, 56.1% of the schoolchildren examined were affected by the combined STH (together *A. lumbricoides*, *T. trichiura,* and Hookworms). This confirms the important level of STH in Central Africa, as in Cameroun [53]. Factors that may explain high levels of STH infections include lacks of sanitation and access to drinking water [9]. Our results indicate that the North region (58.1%) was most prevalent that the East (53.1%) and schoolchildren from rural schools (72.1%) were more affected than those from urban schools (51.6%).Various factors, such as genetics, poly-parasitism, demography, and urbanization, may explain these differences [11]. The most common STH was *T. trichiura* (43.7%), followed by *A. lumbricoides* (35.6%), with heterogeneous distributions between departments (Figure 3) and between schools. Indeed, *T. trichiura* and *A. lumbricoides* were moderately prevalent (≥20 and <50%) in 21 and 19 schools, respectively, and both were very highly prevalent (≥50%) in 16 schools. In contrast to the high prevalence of *A. lumbricoides* and *T. trichiura*, the prevalence of hookworm was low, 1.4% at overall, 1.6% in the North, and 1.2% in the East region. These results confirm the well documented observation that the prevalence of *T. trichiura* and *A. lumbricoides* were always higher than prevalence of hookworms [12,25,26]. Besides prevalence, the intensity of infection is a good indicator for epidemiology of STH. Indeed, most of the morbidity is accounted for by infected individuals who are the most heavily infected [54]. Our results showed a moderate-intensity infections for *T. trichiura*. (1143.2 epg overall) and *A. lumbricoides* (overall 9586.6 epg) and light-intensity infection for hookworms (overall 618 epg). However, 2.1% and 3.4% of schoolchildren had heavy-intensity of infections for *T. trichiura* and *A. lumbricoides,* respectively, attesting the burden of these STH in the surveyed foci.

One of the objectives of our study was to address recommendations for SCH and STH preventive chemotherapy in Gabon. Following WHO guidelines, based on prevalence and intensity of infections, the program is classifying communities according to three strategies: (1) a high prevalence (≥50% for both Schistosomiasis and STH) or heavy-intensity infections, schoolchildren are treated every year, and high risk groups, such as fishermen, are treated; (2) a moderate prevalence (≥10% for Schistosomiasis and ≥20% for STH, but <50% for both schistosomiasis and STH) and light-intensity infections, schoolchildren are treated once every two years; and (3) a low prevalence (<10% for Schistosomiasis and <20% for STH) and light-intensity infections, chemotherapy is made available in health facilities for treatment of suspected cases [15]. For schistosomiasis, considering the low prevalence recorded in our study, we recommend PC of SAC twice during primary schooling (once on entry, again on leaving) for eight departments and individual treatment for the confirmed cases in the Zadié department. We also recommend the diagnosis of other communities at high risk (such as pre-schoolchildren, pregnant women, and special occupation groups) and chemotherapy will be made available in health facilities for treatment of suspected cases according to OMS guidelines [15]. WHO recommended the drug Praziquantel (PZQ) with a dosage of 40mg/Kg for the treatment of schistosomiasis in PTC. Impact of treatment varies according to region and treatment strategy. An annual treatment strategy has significantly reduced prevalence of schistosomiasis 1, 2, and 3 years post-treatment in West Africa, i.e., Burkina Faso [38], Niger [55], Ghana [56]; East Africa i.e., Uganda [57], and in Central Africa, i.e., Cameroun [23,26]. For STH, we recommend a biannual PC strategy including pre and SAC, women of child bearing age including pregnant women in the 2nd and 3rd trimesters and lactating women and adults at high risk in certain occupations (e.g. tea-pickers and miners) for the six departments (Ntem, Haut-Ntem, Haut-Komo, Okano, Mvoung and Zadié), where the prevalence was high (≥50%) and an annual PC strategy for the three departments (Woleu, Ivindo, Lopé) with moderate prevalence. Four anthelminthics are currently on the WHO model list of essential medicines for the treatment and control of STH: albendazole, mebendazole, levamisole, and pyrantel-pamoate [15]. Impact of these different drugs on STH are discussed by Keizer and Utzinger [58]. For these authors, oral single-doses of these drugs show high cure rates against *A. lumbricoides*, but not always against *T trichiura* and hookworms. Combination of mebendazole and levamisole shows the best cure rate against STH [59]. Furthermore, considering the total costs per child treated against schistosomiasis and STH, including drug and delivery, US$ 0.32 in Burkina Faso [35], the PC should integrate and progress with both schistosomiasis and STH.

## Figures and Tables

**Table 1:** Percentage of infected schoolchildren (prevalence) [95% confidence intervals], at overall level, at regional level, according to gender, school area, and school category.

	N	Schistosomiasis			Soil Transmittted Helminthiasis			SCH	STH	SCH-STH
		*S. haematobium*	*S. mansoni*	*S. guineensis*	*A. lumbricoides*	*T. trichiura*	Hookworms			
*Overall*	2245	1.65 [1.16–2.27]	0.04 [0.0–0.25]	0.04 [0.0–0.25]	35.59 [33.61–37.59]	43.74 [41.68–45.82]	1.43 [0.98–2.01]	1.69 [1.2–2.32]	56.08 [54.0–58.15]	56.57 [54.49–58.63]
*By region*										
Northern	1236	1.46 [0.87–2.29]	0.08 [0.0–0.45]	0.08 [0.0–0.45]	29.13 * [26.60–31.75]	52.83 * [50.0–55.65]	1.62 [0.99–2.49]	1.54 [0.93–2.39]	58.09 * [55.29–60.86]	58.5 * [55.69–61.26]
Eastern	1009	1.88 [1.14–2.93]	0.00 [0.0–0.37]	0.00 [0.0–0.37]	43.51 [40.42–46.63]	32.61 [29.72–35.60]	1.19 [0.62–2.07]	1.88 [1.14–2.93]	53.62 [50.48–56.73]	54.2 [51.08–57.32]
*By gender*										
Girl	1116	1.25 [0.69–2.1]	0.00 [0.0–0.33]	0.09 [0.0–0.5]	34.14 [31.36–37.01]	42.47 [39.55–45.43]	0.81 [0.37–1.53]	1.34 [0.75–2.2]	54.84 [51.87–57.79]	55.11 [52.13–58.05]
Boy	1129	2.04 [1.30–3.04]	0.09 [0.0–0.49]	0.00 [0.0–0.49]	37.02 [34.20–39.92]	45.00 [42.07–47.95]	2.04 * [1.30–3.04]	2.04 [1.30–3.04]	57.31 [54.36–60.21]	58.02 [55.08–60.91]
*By school area*										
urban	1754	1.48 [0.97–2.16]	0.06 [0.0–0.32]	0.06 [0.0–0.32]	30.84 [28.69–33.06] *	40.59 [38.28–42.93] *	1.37 [0.88–2.03]	1.54 [1.02–2.23]	51.60 [49.23–53.96] *	52.17 [49.80–54.48] *
rural	491	2.24 [1.12–3.97]	0.00 [0.0–0.75]	0.00 [0.0–0.75]	52.55 [48.02–57.04]	54.99 [50.47–59.45]	1.63 [0.71–3.19]	2.24 [11.24–3.97]	72.10 [67.90–76.02]	72.51 [68.33–76.41]
*By school category*										
Public	1420	1.97 [1.31–2.84]	0.07 [0.0–0.39]	0.07 [0.0–0.39]	36.76 [34.25–39.33]	45.35 [43.74–47.98] *	1.62 [1.03–2.42]	2.04 [1.37–2.92]	57.61 [54.99–60.19]	58.24 [55.62–60.82] *
Private	825	1.09 [0.50–2.06]	0.00 [0.0–0.45]	0.00 [0.0–0.45]	33.58 [30.36–36.91]	40.97 [37.59–44.41]	1.09 [0.50–2.06]	1.09 [0.50–2.06]	53.46 [49.98–56.90]	53.82 [50.35–57.26]

* *p* < 0.05 (Chi squared test), SCH = combined Schistosomiasis (together *S. haematobium*, *S. mansoni* and *S. guineensis*); STH = combined soil transmitted helminthiasis (together *A. lumbricoides*, *T. trichiura* and Hookworms). SCH-STH = presence at least one schistosomiasis and/or soil transmitted helminthiasis.

**Table 2:** Proportion of polyparasitism for schistosomiaisis, soil transmitted helminthiasis (STH) and combined (schistosomiais-STH). N is the total number of examined schoolchildren.

Number of Species	N	Schistosomiasis	STH	Schistosomiasis-STH
1	2245	37	723	718 *
2		1	528	537
3		0	8	15
4		0	0	0
5		0	0	0
6		0	0	0
>6		0	0	0
Negative		2207	986	975

* In the STH column some of those also have schistosomiasis, so this decreases the number with 1 species in the SCH-STH column.

**Table 3:** Mean intensity of infection ± standard deviation at overall, by region, gender, school area, and school category. (N).

	Schistosomiasis			Soil Transmittted Helminthiasis		
	*S. haematobium*	*S. mansoni*	*S. guineensis*	*A. lumbricoides*	*T. trichiura*	Hookworms
*Overall*	101.9 ± 45.1 (37)	72 (1)	240 (1)	9586.6 ± 618.3 (799)	1143.2 ± 97.0 (982)	618.0 ± 499.6 (32)
*By region*						
North	18.3 ± 8.2 (18)	72 (1)	240 (1)	11433.6 ± 1061.7 (360)	1395.2 ± 126.6 (653)	130.8 ± 31.1 (20)
East	181.1 ± 85.6 (19)	-	-	8071.9 ± 707.3 (439)	642.9 ± 140.2 (329)	1430.0 ± 1369.1 (12)
*By gender*						
Girl	65.3 ± 32.4 (14)	-	240 (1)	10131.3 ± 1053.6 (381)	1152.6 ± 147.4 (474)	1861.3 ± 1853.7 (9)
Boy	124.2 ± 70.9 (23)	72 (1)	-	9018.1 ± 688.7 (418)	1130.1 ± 127.7 (508)	131.5 ± 35.0 (23)
*By school area*						
urban	109.5 ± 61.0 (26)	72 (1)	240 (1)	9144.6 ± 665.8 (541)	1139.9 ± 113.9 (712)	791.0 ± 669.2 (24)
rural	174.7 ± 55.3 (11)	-	-	10513.4 ± 1313.1 (258)	1151.7 ± 185.9 (270)	99.0 ± 46.9 (8)
*By school category*						
Public	73.3 ± 26.3 (28)	72 (1)	240 (1)	8874.4 ± 624.4 (522)	1193.5 ± 113.1 (644)	97.0 ± 18.9 (23)
Private	504.9 ± 178.5 (9)	-	-	10928.7 ± 1340.5 (277)	1047.3 ± 182.1 (338)	1949.3 ± 1843.5 (9)

**Table 4:** Diagnosis of health department and recommended treatment strategies.

Department	Category	MDA Interventions in Schools (Enrolled and Non-Enrolled)	Drug
**Schistosomiasis infections**			
Woleu	Low prevalence	MDA of SAC twice during primary schooling (once on entry, again on leaving)	Praziquantel
Ntem			
Haut-Ntem			
Haut-Okano			
Okano			
Ivindo			
Lopé			
Mvoung			
Zadié	Not endemic	No required MDA. Treatment of individual confirmed cases	
**Soil Transmitted Helminthiasis infections**			
Woleu	Moderate prevalence and moderate intensity	Annual MDA	Mebendazole + Levamisole
Ntem	High prevalence	Biannual MDA	
Haut-Ntem			
Haut-Komo			
Okano			
Ivindo	Moderate prevalence and moderate intensity	Annual MDA	
Lopé			
Mvoung	High prevalence	Biannual MDA	
Zadié			

MDA = mass drug administration; SAC = school-aged children.

## Supplementary Materials

The following are available at http://www.mdpi.com/2414-6366/3/4/119/s1, Table S1: list of schools surveyed per region and department with their respective geographical position. Non-italic = public school; italics = private school. In bold = urban school; normal = rural school, Table S2: Number of infected schoolchildren (prevalence in %) for each parasite according to school and department investigated. N is the number of schoolchildren examined. * *p* < 0.05 (Fisher-Exact-test); * is followed by school number or by department name with a significant difference, Table S3: Intensity of infection (mean ± standard deviation) for each parasite according to school and department investigated. N is the number of schoolchildren examined. * *p* < 0.05 (Mann-Whitney test); * is followed by the school number or department code with significant difference. L, M and H indicate class intensity of infection. L = light-intensity infection, M = moderate-intensity infections, H = heavy-intensity infections according to each species.
