# Supplementary material for: Baseline Mapping of Schistosomiasis and Soil Transmitted Helminthiasis in the Northern and Eastern Health Regions of Gabon, Central Africa: Recommendations for Preventive Chemotherapy
Source: Trop Med Infect Dis. 2018 Nov 11;3(4):119. doi: 10.3390/tropicalmed3040119 (PMC6306699; doi:10.3390/tropicalmed3040119)
Supplement: Supplementary file 1 [file tropicalmed-03-00119-s001.zip › Table S3.docx]

S3 Table

| Département | Ecole | Schistosomiasis | | | Soil-Transmitted-Helminthiasis | | |
| --- | --- | --- | --- | --- | --- | --- | --- |
|  |  | *S. haematobium* | *S. mansoni* | *S. guineensis* | *A. lumbricoides* | *T. trichiura* | Hookworm |
| Woleu  (WLE) | 01 | No infection | No infection | No infection | 13419.0±6426.6 (8)^M^ | 1654.9±621.4 (22) ^M^ *2-3-4 | 56.0±8.0 (3) ^L^ |
|  | 02 | 22.0 (1) ^L^ |  |  | 21196.0±7185.6 (12)^M^ | 758.4±336.2 (20) ^L^ *1 | 144.0 (1) ^L^ |
|  | 03 | No infection |  |  | 8872.0±2129.1 (3)^M^ *5 | 276.0±104.7 (14) ^L^ *1 | No infection |
|  | 04 | No infection |  |  | 11048.0±2440.3 (3)^M^ | 507.0±173.0 (16) ^L^ *1 | 240.0±148.0 (4) ^L^ |
|  | 05 | 1.0 (1) ^L^ |  |  | 17712.0±3194.8 (6)^M^ *3 | 1155.3±286.4 (29) ^M^ | 92.6±24.8 (7) ^L^ |
|  | **Total** | **11.5±10.5 (2)** **^L^** |  |  | **16491.8±3194.8 (32)^M^**  ***HKO-OKO** | **961.0±961.0 (101)** **^L^** | **128.0±41.6 (15) ^L^** |
|  | F | 1.0 (1) ^L^ |  |  | 6850.0±1642.7 (12) ^M^ * | 648.0±130.3 (49) ^L^ | 80.0±32.0 (3) ^L^ |
|  | M | 22.0 (1) ^L^ |  |  | 22276.8±4583.7 (20) ^M^ | 1255.9±319.2 (52) ^M^ | 140.0±51.4 (12) ^L^ |
| Ntem  (NTM) | 01 | No infection | No infection | No infection | 15690.0±4270.9 (16) ^M^ | 2388.6±475.9 (19) ^M^ *4 | No infection |
|  | 02 | No infection | No infection |  | 60500.6±30103.5 (7) ^M^ | 6016.0±1944.1 (15) ^M^ | No infection |
|  | 03 | No infection | No infection |  | 12268.8±5744.5 (5) ^M^ | 1592.7±870.5 (11) ^M^ | 216.0 (1) ^L^ |
|  | 04 | 10.7±5.8 (3) ^L^ | 72.0 (1) |  | 9156.0±3811.9 (22) ^M^ | 1263.1±471.6 (54) ^M^ *1-5 | 96.0 (1) ^L^ |
|  | 05 | 28.0±25.5 (3) ^L^ | No infection |  | 8554.8±3929.9 (20) ^M^ | 3221.7±837.8 (34) ^M^ *4 | 240.0 (1) ^L^ |
|  | **Total** | **19.3±12.3 (6)** **^L^** | **72.0 (1)** |  | **15834.5±3854.3 (70) ^M^**  ***OKO** | **2487.9±390.3 (133)** **^M^**  ***HKO-OKO** | **184.0±44.5 (3) ^L^** |
|  | F | 22.8±18.9 (4) ^L^ | No infection |  | 22810.9±7596.7 (33) ^M^ | 2612.2±577.9 (63) ^M^ | 96.0 (1) ^L^ |
|  | M | 12.5±8.5 (2) ^L^ | 72.0 (1) |  | 9612.3±2415.4 (37) ^M^ | 2376.0±532.0 (70) ^M^ | 228.0±12.0 (2) ^L^ |
| Haut-Ntem (HNT) | 01 | 1.0 (1) | No infection | No infection | 15929.7±4628.7 (23) ^M^ *5 | 3409.6±1302.0 (30) ^M^ *4-5 | No infection |
|  | 02 | No infection |  |  | 12436.4±2771.9 (33) ^M^ *5 | 1803.2±317.4 (45) ^M^ *4-5 | 36.0±12.0 (2) ^L^ |
|  | 03 | 13.0 (1) ^L^ |  |  | 12953.3±4315.6 (18) ^M^ | 1465.7±678.8 (29) ^M^ | No infection |
|  | 04 | No infection |  |  | 15150.7±5797.3 (18) ^M^ | 655.7±215.2 (28) ^L^ *1-2 | No infection |
|  | 05 | No infection |  |  | 3288.0±1303.1 (12) ^L^ *1-2 | 370.1±84.9 (19) ^L^ *1-2 | No infection |
|  | **Total** | **7.0±6.0 (2)** **^L^** |  |  | **12712.6±1847.9 (104) ^M^**  ***HKO-OKO** | **1664.4±314.4 (151)** **^M^ *OKO** | **72.0±48.0 (2)** **^L^** |
|  | F | 1.0 (1) ^L^ |  |  | 13805.2±3028.8 (46) ^M^ | 1244.4±408.8 (74) ^M^ * | 24.0 (1) ^L^ |
|  | M | 13.0 (1) ^L^ |  |  | 11846.1±2300.0 (58) ^M^ | 2068.1±473.4 (77) ^M^ | 120.0 (1) ^L^ |
| Haut-Komo (HKO) | 01 | 82.0±47.0 (2) ^L^ | No infection | 240.0 (1) | 6800.0±1442.1 (45) ^M^ | 960.0±243.9 (62) ^L^ | No infection |
|  | 02 | 1.0 (1) ^L^ |  | No infection | 9201.0±4666.2 (16) ^M^ | 656.8±196.9 (30) ^L^ |  |
|  | 03 | No infection |  | No infection | 9801.0±3625.8 (8) ^M^ | 2448.0±1829.5 (14) ^M^ |  |
|  | 04 | No infection |  | No infection | 15097.6±4754.4 (15) ^M^ | 1408.0±519.4 (24) ^M^ |  |
|  | 05 | 1.0 (1) ^L^ |  | No infection | 4903.6±1626.0 (22) ^L^ | 693.2±199.3 (26) ^L^ |  |
|  | **Total** | **33.4±24.8 (5)** **^L^** |  | **240.0 (1)** | **8169.5±1240.4 (106) ^M^**  ***WLE-HTN** | **1059.7 ±211.5 (156)** **^M^ *NTM** |  |
|  | F | 1.0 (1) ^L^ |  | 240.0 (1) | 7919.6±1736.9 (60) ^M^ | 1013.5±209.2 (88) ^M^ |  |
|  | M | 41.5±30.3 (4) ^L^ |  | No infection | 8495.5±1763.5 (46) ^M^ | 1119.5±404.6 (68) ^M^ |  |
| Okano  (OKO) | 01 | 7.0 (1) ^L^ | No infection | No infection | 2905.5±1354.5 (16) ^L^ *3 | 747.2±272.1 (53) ^L^ | No infection |
|  | 02 | No infection |  |  | 6758.0±3181.4 (12) ^M^ | 526.9±114.3 (22) ^L^ |  |
|  | 03 | No infection |  |  | 11496.0±4512.2 (6) ^M^ *1-4 | 413.7±123.1 (17) ^L^ |  |
|  | 04 | 1.0 (1) ^L^ |  |  | 1627.2±680.4 (10) ^L^ *3 | 356.0±99.4 (12) ^L^ |  |
|  | 05 | 2.0 (1) ^L^ |  |  | 12240.0±9369.3 (14) ^M^ | 228.0±86.8 (8) ^L^ |  |
|  | **Total** | **3.3±1.9 (3)** |  |  | **5454.0±1357.3 (48) ^M^**  ***WLE-NTM-HNT** | **574.3±132.9 (112)** **^L^ *NTM-HNT** |  |
|  | F | 1.5±0.5 (2) ^L^ |  |  | 5402.7±2200.8 (18) ^M^ | 603.5±194.5 (55) ^L^ |  |
|  | M | 7.0 (1) ^L^ |  |  | 5484.8±1753.2 (30) ^M^ | 546.1±183.2 (57) ^L^ |  |
| Ivindo  (IVD) | 01 | 35.0 (1) ^L^ | No infection | No infection | 6189.3±3563.2 (9) ^M^ | 977.1±794.2 (14) ^L^ *3-4 | No infection |
|  | 02 | No infection |  |  | 9994.7±3365.4 (18) ^M^ | 144.0±39.9 (16) ^L^ *3-4-5 | 24.0 (1) ^L^ |
|  | 03 | 176.0 (1) ^H^ |  |  | 6890.1±1456.5 (23) ^M^ *5 | 931.0±404.5 (19) ^L^  *1-2-4 | No infection |
|  | 04 | No infection |  |  | 10142.4±2166.3 (40) ^L^ *5 | 365.4±75.3 (31) ^L^ *1-2-3 | No infection |
|  | 05 | No infection |  |  | 891.4±412.5 (7) ^L^ *3-4 | 768.0±480.8 (3) ^L^ *2 | 48.0 (1) ^L^ |
|  | **Total** | **105.5±70.5 (2)** |  |  | **8309.4±1201.3 (97) ^M^**  ***MVG-ZAD** | **569.9±165.7 (83)** **^L^ *LPE-ZAD** | **36.0±12.0 (2)** **^L^** |
|  | F | 35.0 (1) ^L^ |  |  | 8007.6±1892.3 (37) ^M^ | 758.3±404.9 (32) ^L^ * | 36.0 ± 12.0 (2) ^L^ |
|  | M | 176.2 (1) ^H^ |  |  | 8495.6±1564.4 (60) ^M^ | 451.8±93.7 (51) ^L^ | No infection |
| Lopé  (LPE) | 01 | 290.0±276.0 (2) ^H^ | No infection | No infection | 9682.5±2957.7 (32) ^M^ | 184.5±91.5 (16) ^L^ *3 | 88.0±55.0 (6) ^L^ |
|  | 02 | 512.7±510.7 (3) ^H^ |  |  | 12411.8±5354.7 (19) ^M^ | 192.0±42.9 (18) ^L^ | No infection |
|  | 03 | No infection |  |  | 11199.3±4497.8 (11) ^M^ *5 | 363.4±85.2 (7) ^L^ *1-5 | No infection |
|  | 04 | 400.0 (1) ^H^ |  |  | 7153.2±2525.3 (20) ^M^ | 275.1±79.7 (13) ^L^ | 168.0 (1) ^L^ |
|  | 05 | No infection |  |  | 1952.0±818.4 (12) ^M^ *3 | 195.7±90.5 (13) ^L^ *3 | No infection |
|  | **Total** | **419.7±243.4 (6)** **^H^** |  |  | **8886.6±1662.9 (94)** **^M^** | **225.0±34.8 (67)** **^L^**  ***IVD-MVG-ZAD** | **99.4±47.8 (7)** **^L^** |
|  | F | 400.0 (1) ^H^ |  |  | 7248.0±2026.4 (45) ^M^ | 163.4±208.7 (31) ^L^ | 264.0±96.0 (2) ^L^ |
|  | M | 423.6±298.0 (5) ^H^ |  |  | 10391.5±2592.8 (49) ^M^ | 278.0±331.3 (36) ^L^ | 33.6±9.6 (5) ^L^ |
| Mvoung (MVG) | 01 | 84.0±77.1 (3) ^L^ | No infection | No infection | 1576.5±500.6 (16) ^M^ *3 | 341.7±119.7 (17) ^L^ *3 | No infection |
|  | 02 | 60.5±52.5 (2) ^L^ |  |  | 13016.0±6773.9 (18) ^M^ | 2304.0± 1743.2 (8) ^M^ | 5464.0±5190.0 (3) ^H^ |
|  | 03 | No infection |  |  | 6831.0±1397.8 (48) ^M^ *1 | 1603.5±654.1 (58) ^M^ *1-5 | No infection |
|  | 04 | No infection |  |  | No infection | 24.0 (1) ^L^ | No infection |
|  | 05 | 56.5±37.9 (6) ^L^ |  |  | 12240.0±5886.9 (19) ^M^ | 1296.0± 1118.9 (13) ^M^ *3 | No infection |
|  | **Total** | **64.7±27.9 (11)** **^L^** |  |  | **8118.4±1776.6 (101) ^M^ *IVD** | **1382.6±441.4 (97)** **^M^ *LPE-ZAD** | **5464.0±5190.0 (3)** **^L^** |
|  | F | 91.5±54.8 (4) ^L^ |  |  | 10431.6±3116.2 (54) ^M^ | 2368.8±1038.8 (40) ^M^ | 528.0 (1) ^L^ |
|  | M | 49.4±32.8 (7) ^L^ |  |  | 5460.8±1266.3 (47) ^M^ | 690.5±141.9 (57) ^L^ | 7932.0±7908.0 (2) ^L^ |
| Zadié  (ZAD) | 01 | No infection | No infection | No infection | 4144.6±993.0 (42) ^L^ *2-5 | 231.7±164.6 (26) ^L^ | No infection |
|  | 02 |  |  |  | 13638.4±3425.6 (41)^L^ *1-3 | 108.7±31.9 (17) ^L^ |  |
|  | 03 |  |  |  | 4252.4±1111.13 (33) ^L^ *2 | 132.0 ±61.8 (22) ^L^ |  |
|  | 04 |  |  |  | 8462.4±4497.6 (5) ^M^ | 992.0 ± 944.0 (3) ^L^ |  |
|  | 05 |  |  |  | 6397.9±1412.9 (26) ^M^ *1 | 90.9±23.9 (14) ^L^ |  |
|  | **Total** |  |  |  | **7362.1± 1108.0 (147) ^M^**  ***IVD** | **183.2±64.2 (82)** ^L^  ***IVD-LPE-MVG** |  |
|  | F |  |  |  | 8313.8±1855.1 (76) **^M^** | 272.6±120.3 (42) ^L^ |  |
|  | M |  |  |  | 6343.4±1151.4 (71) **^M^** | 89.4±33.8 (40) ^L^ |  |
