# Supplementary material for: Baseline Mapping of Schistosomiasis and Soil Transmitted Helminthiasis in the Northern and Eastern Health Regions of Gabon, Central Africa: Recommendations for Preventive Chemotherapy
Source: Trop Med Infect Dis. 2018 Nov 11;3(4):119. doi: 10.3390/tropicalmed3040119 (PMC6306699; doi:10.3390/tropicalmed3040119)
Supplement: Supplementary file 1 [file tropicalmed-03-00119-s001.zip › Table S1.docx]

S1 Table

| **Region** | **Department** | **School** | **School name** | **GPS point** |
| --- | --- | --- | --- | --- |
| Nord | Woleu | 1 | **Ecole Provinciale d’Oyem** | 01.59967°N  011.56645°E |
|  |  | 2 | ***Ecole Privée Protestante André Mintsa*** | 01.59049°N  011.57013°E |
|  |  | 3 | ***Ecole Privée Catholique St Eloi*** | 01.61000°N  011.57861°E |
|  |  | 4 | ***Ecole Privée Catholique St Macaire*** | 01.61189°N  011.57761°E |
|  |  | 5 | **Ecole Publique Communale de Methui** | 01.60977°N  011.58814°E |
|  | Ntem | 1 | Ecole Publique Rurale de Konoville | 01.84280°N  011.64474°E |
|  |  | 2 | *Ecole Privée Protestante D’Awoua* | 01.87748°N  011.66107°E |
|  |  | 3 | Ecole Publique Rurale d’Akam-Effak | 01.93242°N  011.58912°E |
|  |  | 4 | **Ecole Publique Communale de Bitam Centre** | 02.07847°N  011.49518°E |
|  |  | 5 | **Ecole Publique Communale d’Agnizok** | 02.08226°N  011.48370°E |
|  | Haut Ntem | 1 | **Ecole Communale B (Ngone)** | 02.13607°N  012.14867°E |
|  |  | 2 | **Ecole Communale A** | 02.15187°N  012.13923°E |
|  |  | 3 | **Ecole Communale C (Ngame)** | 02.14739°N  012.13128°E |
|  |  | 4 | *Ecole Privée Protestante d’Ebomane* | 02.16718°N  012.06080°E |
|  |  | 5 | *Ecole Privée Catholique d’Alep* | 02.15608°N  012.08179°E |
|  | Haut Komo | 1 | **Ecole Communale Publique de Medouneu** | 01.01097°N  010.79521°E |
|  |  | 2 | ***Ecole Privée Catholique St J Batiste*** | 01.01779°N  010.80172°E |
|  |  | 3 | *Ecole Privée Catholique de NkomeMbe* | 00.99733°N  010.71691°E |
|  |  | 4 | Ecole Publique de Nkinen’n | 00.96235°N  010.6855°E |
|  |  | 5 | Ecole Publique de Komo Aviation | 01.00371°N  010.76233°E |
|  | Okano | 1 | **Ecole Publique Communale de Mitzic Centre** | 00.78262°N  011.54924°E |
|  |  | 2 | ***Ecole Privée Catholique St Joseph*** | 00.79213°N  011.55108°E |
|  |  | 3 | ***Ecole Privée Protestante de Mitzic Centre*** | 00.78497°N  011.54987°E |
|  |  | 4 | Ecole Publique Rurale de Doumandzou | 00.95037°N  011.15754E |
|  |  | 5 | Ecole Publique de MinkoMibè | 00.86721°N  011.58973°E |
| Est | Ivindo | 1 | ***Ecole Privée Catholique Notre Dame des Victoires*** | 00.56826°N  012.85146°E |
|  |  | 2 | **Ecole Publique de MboloEdock** | 00.55421°N  012.85069°E |
|  |  | 3 | **Ecole Publique Communale de Loaloa** | 00.52809°N  012.82878°E |
|  |  | 4 | Ecole Publique de Mohoba-Mozeve | 00.31776°N  013.22416°E |
|  |  | 5 | *Ecole Privée Protestante de Mbess* | 00.57652°N  012.68767°E |
|  | Lopé | 1 | Ecole publique rurale de Djidji | 00.23061°N  011.79161°E |
|  |  | 2 | ***Ecole Saint Marcel de Massoukou*** | 00.07734°S  011.94719°E |
|  |  | 3 | **Ecole Publique de Tsong-Bial** | 00.11018°S  011.94016°E |
|  |  | 4 | **Ecole Publique Communale de Booué Centre** | 00.09289°S  011.94747°E |
|  |  | 5 | Ecole Publique de Petit Okano (Abénéland) | 00.04751°S  011.87439E |
|  | Mvoung | 1 | **Ecole Publique Communale d’Ovan** | 00.31258°N  012.17812°E |
|  |  | 2 | ***Ecole Privée protestante d’Ovan*** | 00.30760°N  012.18476°E |
|  |  | 3 | ***Ecole Communale Privée Protestante de Kombani*** | 00.32602°N  012.19575°E |
|  |  | 4 | Ecole Publique de Mintoum | 00.41891°N  012.26924°E |
|  |  | 5 | Ecole Publique de Koumameyong | 00.21880°N  011.87133°E |
|  | Zadié | 1 | **Ecole Publique Communale B de Mékambo** | 00.99936°N  013.95943°E |
|  |  | 2 | ***Ecole Privée protestante de Mékambo*** | 01.01200°N  013.94155°E |
|  |  | 3 | ***Ecole P. Catholique Notre Dame des Rosaires*** | 01.00863°N  013.96477°E |
|  |  | 4 | *Ecole Privée Catholique d’Imbong* | 01.03461°N  013.99459°E |
|  |  | 5 | Ecole Publique Rurale de Malassa | 00.90206°N  014.04312°E |
