# Supplementary material for: Baseline Mapping of Schistosomiasis and Soil Transmitted Helminthiasis in the Northern and Eastern Health Regions of Gabon, Central Africa: Recommendations for Preventive Chemotherapy
Source: Trop Med Infect Dis. 2018 Nov 11;3(4):119. doi: 10.3390/tropicalmed3040119 (PMC6306699; doi:10.3390/tropicalmed3040119)
Supplement: Supplementary file 1 [file tropicalmed-03-00119-s001.zip › Table S2.docx]

**S2 Table**

| Department | School | N | Schistosomiasis | | | | Soil-Transmitted-Helminthiasis | | | | SCH-STH |
| --- | --- | --- | --- | --- | --- | --- | --- | --- | --- | --- | --- |
|  |  |  | *S. haematobium* | *S. mansoni* | *S. guineensis* | SCH | *A. lumbricoides* | *T. trichiura* | Hookworm | STH |  |
| Woleu (WLE) | 1 | 50 | 0 (0.0) | 0 (0.0) | 0 (0.0) | 0 (0) | 8 (16.0) | 22 (44.0) | 3 (6.0) | 24 (48.0) | 24 (48.0) |
|  | 2 | 50 | 1 (2.0) | 0 (0.0) | 0 (0.0) | 1 (2.0) | 12 (24.0) *3-4 | 20 (40.0) | 1 (2.0) *5 | 25 (50.0) *3 | 26 (52.0) *3 |
|  | 3 | 49 | 0 (0.0) | 0 (0.0) | 0 (0.0) | 0 (0) | 3 (6.1) *2 | 14 (28.6) *5 | 0 (0.0) *5 | 14 (28.6) *2-5 | 14 (28.6) *2-5 |
|  | 4 | 50 | 0 (0.0) | 0 (0.0) | 0 (0.0) | 0 (0) | 3 (6.0) *2 | 16 (32.0) *5 | 4 (8.0) | 17 (34.0) *5 | 17 (34.0) *5 |
|  | 5 | 49 | 1 (2.0) | 0 (0.0) | 0 (0.0) | 1 (2.0) | 6 (12.2) | 29 (59.2) *3-4 | 7 (14.3) *2-3 | 30 (61.2) *3-4 | 30 (61.2) *3-4 |
|  | **Total** | **248** | **2 (0.8)** | **0 (0)** | **0 (0)** | **2 (0.8)** | **32 (12.9)**  ***NTM-HNT-HKO** | **101 (40.7) *NTM-HNT-HKO** | **15 (6.0)**  ***NTM-HNT-HKO-OKO** | **110 (44.4) *NTM-HNT-HKO** | **111 (44.8)**  ***NTM-HNT-HKO** |
|  | F | 124 | 1 (0.8) | 0 (0.0) | 0 (0.0) | 1 (0.8) | 12 (9.7) | 49 (39.5) | 3 (2.4) * | 51 (41.1) | 51 (41.1) |
|  | M | 124 | 1 (0.8) | 0 (0.0) | 0 (0.0) | 1 (0.8) | 20 (16.1) | 52 (41.9) | 12 (9.7) | 59 (47.6) | 60 (48.4) |
| Ntem  (NTM) | 1 | 25 | 0 (0.0) | 0 (0.0) | 0 (0.0) | 0 (0) | 16 (64.0) *4-5 | 19 (76.0) *4-5 | 0 (0.0) | 20 (80.0) *4-5 | 20 (80.0) *4-5 |
|  | 2 | 17 | 0 (0.0) | 0 (0.0) | 0 (0.0) | 0 (0) | 7 (41.2) *4 | 15 (88.2) *4-5 | 0 (0.0) | 15 (88.2) *4-5 | 15 (88.2) *4-5 |
|  | 3 | 14 | 0 (0.0) | 0 (0.0) | 0 (0.0) | 0 (0) | 5 (35.7) | 11 (78.6) *4 | 1 (7.1) | 13 (92.9) *4-5 | 13 (92.9) *4-5 |
|  | 4 | 124 | 3 (2.4) | 1 (0.8) | 0 (0.0) | 3 (2.4) | 22 (17.7) *1-2 | 54 (43.5) *1-2-3 | 1 (0.8) | 61 (49.2) | 62 (50.0) *1-2-3 |
|  | 5 | 69 | 3 (4.3) | 0 (0.0) | 0 (0.0) | 3 (4.3) | 20 (29.0) *1 | 34 (49.3) *1-2 | 1 (1.4) | 36 (52.2) | 38 (55.1) *1-2-3 |
|  | **Total** | **249** | **6 (2.4)** | **1 (0.4)** | **0 (0.0)** | **6 (2.4)** | **70 (28.1)**  *** HNT-HKO-OKO** | **133 (53.4) *HKO** | **3 (1.2)** | **145 (58.2) * HKO-OKO** | **148 (59.4) * HKO-OKO** |
|  | F | 125 | 4 (3.2) | 0 (0.0) | 0 (0.0) | 4 (3.2) | 33 (26.4) | 63 (50.4) | 1 (0.8) | 65 (52.0) | 69(55.2) |
|  | M | 124 | 2 (1.6) | 1 (0.8) | 0 (0.0) | 2 (1.6) | 37 (29.8) | 70 (56.5) | 2 (1.6) | 80 (64.5) | 79 (63.7) |
| Haut-Ntem (HNT) | 1 | 45 | 1 (2.2) | 0 (0.0) | 0 (0.0) | 1 (2.2) | 23 (51.1) *3 | 30 (66.7) *3 | 0 (0.0) | 32 (71.1) *3 | 32 (71.1) *3 |
|  | 2 | 74 | 0 (0.0) | 0 (0.0) | 0 (0.0) | 0 (0) | 33 (44.6) *3 | 45 (60.8) *3 | 2 (2.7) | 55 (74.3) *3 | 55 (74.3) *3 |
|  | 3 | 68 | 1 (1.5) | 0 (0.0) | 0 (0.0) | 1 (1.5) | 18 (26.5) *1-2 | 29 (42.6) *1-2-4 | 0 (0.0) | 30 (44.2) *4 | 30 (44.2) *4 |
|  | 4 | 43 | 0 (0.0) | 0 (0.0) | 0 (0.0) | 0 (0) | 18 (41.9) | 28 (65.1) *3 | 0 (0.0) | 33 (76.7) | 33 (76.7) |
|  | 5 | 30 | 0 (0.0) | 0 (0.0) | 0 (0.0) | 0 (0) | 12 (40.0) | 19 (63.3) | 0 (0.0) | 20 (66.7) | 20 (66.7) |
|  | **Total** | **260** | **2 (0.8)** | **0 (0.0)** | **0 (0.0)** | **2 (0.8)** | **104 (40.0) *OKO** | **151 (58.1) *HKO-OKO** | **2 (0.8)** | **170 (65.4) *OKO** | **170 (65.4)**  ***OKO** |
|  | F | 137 | 1 (0.7) | 0 (0.0) | 0 (0.0) | 1 (0.7) | 46 (33.6) * | 74 (54.0) | 1 (0.7) | 82 (59.9) | 82 (59.9) |
|  | M | 123 | 1 (0.8) | 0 (0.0) | 0 (0.0) | 1 (0.7) | 58 (47.2) | 77 (62.6) | 1 (0.8) | 88 (71.5) | 88 (71.5) |
| Haut-Komo (HKO) | 1 | 99 | 2 (2.0) | 0 (0.0) | 1 (1.0) | 3 (3.0) | 45 (45.5) *2-5 | 62 (62.6) *4 | 0 (0.0) | 67 (67.7) *4-5 | 68 (68.7) *4-5 |
|  | 2 | 60 | 2 (3.3) | 0 (0.0) | 0 (0.0) | 2 (3.3) | 16 (26.7) *1-4-5 | 30 (50.0) *3-4-5 | 0 (0.0) | 36 (60.0) *3-4 | 36 (60.0) *3-4-5 |
|  | 3 | 16 | 0 (0.0) | 0 (0.0) | 0 (0.0) | 0 (0) | 8 (50.0) | 14 (87.5) *2 | 0 (0.0) | 14 (87.5) | 14 (87.5) |
|  | 4 | 26 | 0 (0.0) | 0 (0.0) | 0 (0.0) | 0 (0) | 15 (57.7) *2 | 24 (92.3) *1-2 | 0 (0.0) | 24 (92.3) | 24 (92.3) |
|  | 5 | 30 | 1 (3.3) | 0 (0.0) | 0 (0.0) | 1 (3.3) | 22 (73.3) *1-2 | 26 (86.7) *1-2 | 0 (0.0) | 28 (93.3) | 28 (93.3) |
|  | **Total** | **231** | **5 (2.2)** | **0 (0.0)** | **1 (0.4)** | **6 (2.6)** | **106 (45.9) *WLE-NTM-OKO** | **156 (67.5) *WLE-NTM-HNT-OKO** | **0 (0.0)**  ***WLE** | **169 (73.2) *OKO** | **170 (73.6)**  *** OKO** |
|  | F | 122 | 1 (0.8) | 0 (0.0) | 1 (0.8) | 2 (1.6) | 60 (49.2) | 88 (72.1) | 0 (0.0) | 96 (78.7) | 96 (78.7) |
|  | M | 109 | 4 (3.7) | 0 (0.0) | 0 (0.0) | 4 (3.7) | 46 (42.2) | 68 (62.4) | 0 (0.0) | 73 (67.0) | 74 (67.9) |
| Okano  (OKO) | 1 | 104 | 1 (1.0) | 0 (0.0) | 0 (0.0) | 1 (1.0) | 16 (15.4) *4 | 53 (51.0) | 0 (0.0) | 59 (56.7) *3-4 | 59 (56.7) *3-4 |
|  | 2 | 52 | 0 (0.0) | 0 (0.0) | 0 (0.0) | 0 (0) | 12 (23.1) *4 | 22 (42.3) *4 | 0 (0.0) | 24 (46.2) *4 | 24 (46.2) *4 |
|  | 3 | 50 | 0 (0.0) | 0 (0.0) | 0 (0.0) | 0 (0) | 6 (12.0) *4 | 17 (34.0) *4 | 0 (0.0) | 18 (36.0) *4 | 18 (36.0) *1-4 |
|  | 4 | 16 | 1 (6.3) | 0 (0.0) | 0 (0.0) | 1 (6.3) | 10 (62.5) *1-2-3-5 | 12 (75.0) *2-3-5 | 0 (0.0) | 14 (87.5) *5 | 14 (87.5) *5 |
|  | 5 | 26 | 1 (3.9) | 0 (0.0) | 0 (0.0) | 1 (3.9) | 4 (15.4) *4 | 8 (30.8) *4 | 0 (0.0) | 9 (34.6) | 9 (34.6) |
|  | **Total** | **248** | **3 (1.2)** | **0 (0.0)** | **0 (0.0)** | **3 (1.2)** | **48 (19.4)** | **112 (45.2)** | **0 (0.0)** | **124 (50.0)** | **124 (50.0)** |
|  | F | 124 | 1 (0.8) | 0 (0.0) | 0 (0.0) | 1 (0.8) | 18 (14.5) | 55 (44.4) | 0 (0.0) | 59 (47.6) | 59 (47.6) |
|  | M | 124 | 2 (1.6) | 0 (0.0) | 0 (0.0) | 2 (1.6) | 30 (24.2) | 57 (46.0) | 0 (0.0) | 65 (52.4) | 65 (52.4) |
| Ivindo  (IVD) | 01 | 75 | 1 (1.3) | 0 (0.0) | 0 (0.0) | 1 (1.3) | 9 (12.0) *3-4 | 14 (18.7) *3-4 | 0 (0.0) | 17 (22.7) *3-4 | 17 (22.7) *3-4 |
|  | 02 | 70 | 0 (0.0) | 0 (0.0) | 0 (0.0) | 0 (0.0) | 18 (25.7) *3-4 | 16 (22.9) *3-4 | 1 (1.4) | 26 (37.1) *3-4 | 26 (37.1) *3-4 |
|  | 03 | 34 | 1 (2.9) | 0 (0.0) | 0 (0.0) | 1 (2.9) | 23 (67.6) *5 | 19 (55.9) *1-2-5 | 0 (0.0) | 26 (76.5)*5 | 27 (79.4) *5 |
|  | 04 | 51 | 0 (0.0) | 0 (0.0) | 0 (0.0) | 0 (0.0) | 40 (78.4) *5 | 31 (60.8) *1-2-5 | 0 (0.0) | 45 (88.2)*5 | 45 (88.2) *5 |
|  | 05 | 25 | 0 (0.0) | 0 (0.0) | 0 (0.0) | 0 (0.0) | 7 (28.0) | 3 (12.0) *3-4 | 1 (4.0) | 8 (32.0) | 8 (32.0) |
|  | **Total** | **255** | **2 (0.8) *MVG** | **0 (0.0)** | **0 (0.0)** | **2 (0.8) * MVG** | **97 (38.0) *ZAD** | **83 (32.5)** | **2 (0.8)** | **122 (47.8) *ZAD** | **123 (48.2) *ZAD** |
|  | F | 113 | 1 (0.9) | 0 (0.0) | 0 (0.0) | 1 (0.9) | 37 (32.7) | 32 (28.3) | 0 (0.0) | 52 (46.0) | 52 (46.0) |
|  | M | 142 | 1 (0.7) | 0 (0.0) | 0 (0.0) | 1 (0.7) | 60 (42.3) | 51 (35.9) | 2 (1.4) | 70 (49.3) | 71 (50.0) |
| Lopé  LPE) | 01 | 47 | 2 (4.3) | 0 (0.0) | 0 (0.0) | 2 (4.3) | 32 (68.1) *2-3-4-5 | 16 (34.0) *3 | 6 (12.8) *2-4 | 35 (74.5) *2-3-4 | 35 (74.5) *2-3-4 |
|  | 02 | 49 | 3 (6.1) | 0 (0.0) | 0 (0.0) | 3 (6.1) | 19 (38.8) | 18 (36.7) *3-4 | 0 (0.0) *1 | 25 (51.0) *3 | 25 (51.0) *3 |
|  | 03 | 50 | 0 (0.0) | 0 (0.0) | 0 (0.0) | 0 (0.0) | 11 (22.0) | 7 (14.0) *1-2-5 | 0 (0.0) *1 | 12 (24.0) *5 | 12 (24.0) *5 |
|  | 04 | 72 | 1 (1.4) | 0 (0.0) | 0 (0.0) | 1 (1.4) | 20 (27.8) | 13 (18.1) *2-5 | 1 (1.4) *1 | 27 (37.5) | 28 (38.9) |
|  | 05 | 29 | 0 (0.0) | 0 (0.0) | 0 (0.0) | 0 (0.0) | 12 (41.4) | 13 (44.8) *3-4 | 0 (0.0) *1 | 16 (55.2) | 16 (55.2) |
|  | **Total** | **247** | **6 (2.4) *ZAD** | **0 (0.0)** | **0 (0.0)** | **6 (2.4) *ZAD** | **94 (38.1) *ZAD** | **67 (27.1) *MVG** | **7 (2.8) *ZAD** | **115 (46.6) *ZAD** | **116 (47.0) *ZAD** |
|  | F | 122 | 1 (0.8) | 0 (0.0) | 0 (0.0) | 1 (0.8) | 45 (36.9) | 31 (25.4) | 2 (1.6) | 57 (46.7) | 58 (47.5) |
|  | M | 125 | 5 (4.0) | 0 (0.0) | 0 (0.0) | 5 (4.0) | 49 (39.2) | 36 (28.8) | 5 (4.0) | 58 (46.4) | 58 (46.4) |
| Mvoung  (MVG) | 01 | 79 | 3 (3.8) | 0 (0.0) | 0 (0.0) | 3 (3.8) | 16 (20.3) *2-3-5 | 17 (21.5) *3 | 0 (0.0) | 25 (31.7) *3-5 | 27 (34.2) *3-5 |
|  | 02 | 47 | 2 (4.3) | 0 (0.0) | 0 (0.0) | 2 (4.3) | 18 (38.3) *3-4 | 8 (17.0) *3 | 3 (6.4) | 20 (42.6) *3-4 | 21 (44.7) *3-4 |
|  | 03 | 76 | 0 (0.0) *5 | 0 (0.0) | 0 (0.0) | 0 (0.0) | 48 (63.2) *4 | 58 (76.3) *1-2-4-5 | 0 (0.0) | 62 (81.6) *4 | 62 (81.6) *4 |
|  | 04 | 12 | 0 (0.0) | 0 (0.0) | 0 (0.0) | 0 (0.0) | 0 (0.0) *5 | 1 (8.3) *3 | 0 (0.0) | 1 (8.3) *5 | 1 (8.3) *5 |
|  | 05 | 38 | 6 (15.8) *3 | 0 (0.0) | 0 (0.0) | 6 (15.8) | 19 (50.0) | 13 (34.2) *3 | 0 (0.0) | 24 (63.2) | 26 (68.4) |
|  | **Total** | **252** | **11 (4.4)**  ***ZAD** | **0 (0.0)** | **0 (0.0)** | **11 (4.4) *ZAD** | **101 (40.1)**  ***ZAD** | **97 (38.5)**  ***LPE** | **3 (1.2)** | **132 (52.4)**  ***ZAD** | **137 (54.4)**  ***ZAD** |
|  | F | 124 | 4 (3.2) | 0 (0.0) | 0 (0.0) | 4 (3.2) | 54 (43.5) | 40 (32.3) | 2 (1.6) | 60 (48.4) | 62 (50.0) |
|  | M | 128 | 7 (5.5) | 0 (0.0) | 0 (0.0) | 7 (5.5) | 47 (36.7) | 57 (44.5) | 1 (0.8) | 72 (56.3) | 75 (58.6) |
| Zadié  (ZAD) | 01 | 80 | 0 (0.0) | 0 (0.0) | 0 (0.0) | 0 (0.0) | 42 (52.5) | 26 (32.5) | 0 (0.0) | 50 (62.5) | 50 (62.5) |
|  | 02 | 66 | 0 (0.0) | 0 (0.0) | 0 (0.0) | 0 (0.0) | 41 (62.1) | 17 (25.8) | 0 (0.0) | 43 (65.2) | 43 (65.2) |
|  | 03 | 63 | 0 (0.0) | 0 (0.0) | 0 (0.0) | 0 (0.0) | 33 (52.4) | 22 (34.9) | 0 (0.0) | 44 (69.8) | 44 (69.8) |
|  | 04 | 7 | 0 (0.0) | 0 (0.0) | 0 (0.0) | 0 (0.0) | 5 (71.4) | 3 (42.9) | 0 (0.0) | 6 (85.7) | 6 (85.7) |
|  | 05 | 39 | 0 (0.0) | 0 (0.0) | 0 (0.0) | 0 (0.0) | 26 (66.7) | 14 (35.9) | 0 (0.0) | 29 (74.4) | 29 (74.4) |
|  | **Total** | **255** | **0 (0.0)** | **0 (0.0)** | **0 (0.0)** | **0 (0.0)** | **147 (57.6)**  ***IVD-LPE-MVG** | **82 (32.2)** | **0 (0.0)**  ***LPE** | **172 (67.5)** | **172 (67.5)** |
|  | F | 125 | 0 (0.0) | 0 (0.0) | 0 (0.0) | 0 (0.0) | 76 (60.8) | 42 (33.6) | 0 (0.0) | 87 (69.6) | 87 (69.6) |
|  | M | 130 | 0 (0.0) | 0 (0.0) | 0 (0.0) | 0 (0.0) | 71 (54.6) | 40 (30.8) | 0 (0.0) | 85 (65.4) | 85 (65.4) |

**p*<0.05 (Fisher-Exact-test); * was following by school number or by department name with a significant difference; NS: no significant
